# Testing of Action of Direct Flame on Concrete

**DOI:** 10.1155/2015/371913

**Published:** 2015-02-19

**Authors:** Lenka Bodnarova, Jaroslav Valek, Petr Novosad

**Affiliations:** Faculty of Civil Engineering, Brno University of Technology, Institute of Technology of Building Materials and Components, Veveri 331/95, 602 00 Brno, Czech Republic

## Abstract

The paper states results of experimental exposition of concrete test specimens to direct flame. Concrete test specimens made from various mixtures differing in the type of aggregate, binder, dispersed reinforcement, and technological procedure were subjected to thermal load. Physicomechanical and other properties of all test specimens were tested before exposition to open flame: density, compressive strength, flexural strength, moisture content, and surface appearance. The specimens were visually observed during exposition to open flame and changes were recorded. Exposed surface was photographically documented before thermal load and at 10-minute intervals. Development of temperature of the specimens was documented with a thermocamera. After exposition to thermal load and cooling down, concrete specimens were visually observed, network of cracks was photographically documented, and maximal depth of spalled area was measured.

## 1. Introduction

Concrete has many properties which are advantageous from the point of view of fire resistance. It is not combustible and its thermal conductivity is low [1–4]. However, concrete structures, which are not designed with respect to fire resistance, show considerable level of damage after thermal load. First of all, it is explosive spalling, which results in weakening the cross section of given reinforced concrete structure and exposition of reinforcement bars to temperatures [2], which exceed the critical value of reinforcement [5]. Only few means of preventing or moderating the impacts of thermal load of concrete are used. Protection systems can be divided into two larger groups: active and passive [6]. Active systems are designed with the purpose of maximal possible reduction of acting temperatures. Passive systems directly resist to acting high temperatures and fire. One of the passive systems is the design of fire resistant concrete mixture as such, which can be achieved first of all by addition of dispersed reinforcement of polypropylene fibers [2].

As regards action of high temperatures on concrete, there are differences between concrete with common strengths and high performance concrete [2, 3]. Porosity and transportation capabilities of cement matrix of concrete are very important parameters influencing the level of degradation of concrete, apart from moisture content of concrete [3]. Expanded water vapor, which can pass through open pore structure of cement matrix, causes less destruction than water vapor trapped in dense matrix. Influence of pore structure of cement matrix on reduction of destruction effect of expanded water vapor is considerably limited with high performance concrete (HPC), high strength concrete (HSC), and self-compacting concrete (SCC). To achieve required properties of such concrete, superplasticizers and fine grained mineral additions are used. These types of concrete have considerably dense and homogeneous structure of cement matrix with reduced capillary porosity, which significantly reduces diffusion of water vapor [3].

One of the factors contributing to explosive spalling is moisture content [2, 3], because no explosive spalling occurs if the moisture content in concrete is very low. Some laboratory tests are carried out with fully saturated specimens (storing in water bath), which makes the moisture condition comparable for various tests. However, humid, fully saturated concrete does not correspond to real condition of a concrete structure. It is necessary to consider the type of structure in which the given type of concrete should be used and adjust moisture of tested specimens to real moisture conditions of given structure (e.g., moisture of tunnel lining could be around 4.0% [7], in some cases considerably higher, 6-7% [8]).

As regards influence of high temperatures, aggregate has the key role and it should be taken into account from the very start of designing the concrete mixture. Aggregate takes up about 60–80% of its volume and various types of aggregate can have crucial role for resistance of concrete to high temperatures. Individual types of aggregate react differently to high temperatures; both physical and chemical changes may occur during exposition to heat. The key factor of behavior of thermally loaded concrete is chemical and physical stability of aggregate. For this reason, selection of appropriate aggregate is very important for ensuring thermal stability of concrete.

If thermal resistance of concrete should be improved by selection of binder, it is necessary to limit Portland cement and use blended Portland cements and other cements with the lowest possible content of Portland clinker. The goal is the lowest possible content of Portlandite Ca(OH)_2_, which is formed during hydration of cement and decomposes at temperatures around 500°C [4]. Using cement with reduced content of Portland clinker is especially important for structural failure of the hardened cement stone (microcracks, consistency with filler, and cement stone strength) [4]. The amount of Portland clinker does not have crucial influence on the resistance to explosive spalling.

Use of blended Portland cements has also positive ecological impact because it helps reduce CO_2_ emissions and it is also connected with other research works focusing on reduction of CO_2_ emissions during production of binders [9].

## 2. Experimental Works

### 2.1. Selection of Individual Components of Concrete

Test specimens of various compositions for experimental works, loading with open flame, had the shape of cubes with dimensions 100 × 100 × 100 mm. Different mixtures were selected, their composition is given in Table 2; they differed in the type of aggregate, binder, and dispersed reinforcement. The reason for variety of composition of individual mixtures was achieving differences in properties so that influence of the differences on resistance to open flame could be evaluated.

Dose of cement (with the exception of the mixture CEM I - GR-SCC) was identical for all mixtures: 350 kg/m^3^. Mixture CEM I - GR-SCC (360 kg/m^3^ of cement) had increased dose of cement for the reason of achieving higher strengths and self-compacting properties in fresh state. Two cements were used for the purpose of evaluating influence of used type of cement: CEM I 42.5 R (concrete mixtures CEM I - GR-PF, CEM I - GR-SCC) and blast-furnace cement CEM III/B 32.5 N (concrete mixtures CEM III/B - B, CEM III/B - B-PF, and CEM III/B - B-SF).

To evaluate influence of the type of coarse aggregate, two types of aggregate were used: granodiorite, biotite granodiorite (concrete mixtures CEM I - GR-PF, CEM I - GR-SCC) and basalt (concrete mixtures CEM III/B - B, CEM III/B - B-PF, and CEM III/B - B-SF).

Composition of granodiorite aggregate: 42% plagioclase, 34% quartz, 15% spar, 8% biotite, and 1% other [12]. Composition of basalt aggregate: 37–45% pyroxene, 20–30% plagioclase, 10–25% magnetite, 15–20% chrysolite, and 1–3% nepheline [13]. Aggregate basalt was selected for reason of good stability at high temperatures [14].

Two types of aggregate were used for small fraction (0/4 mm). In mixtures CEM I - GR-PF, CEM I - GR-SCC, natural mined, and nonwashed aggregate, spar gravelous sand was used [15]. In mixtures CEM III/B - B, CEM III/B - B-PF, and CEM III/B - B-SF natural crushed basalt aggregate was used [13].

Concrete mixture CEM III/B - B (without fibers) was designed for evaluation of influence of various fibers. This mixture was modified by addition of polypropylene fiber reinforcement, which is recommended for fire resistant concrete by manufacturer, dose 1 kg/m^3^, concrete mixture CEM III/B - B-PF and by addition of fine steel fibers with low content of carbon, also recommended for fire resistant concrete by manufacturer, dose 50 kg/m^3^, and concrete mixture CEM III/B - B-SF. Concrete mixture CEM I - GR-PF used different type of polypropylene reinforcement, made by the same manufacturer as reinforcement used in CEM III/B - B-PF (shorter and thinner fibers) dose was twice higher than recommended, 2 kg/m^3^. Parameters of dispersed reinforcement used, differing in material of fibers and dimensions, are given in Table 1. Increased dose of polypropylene fibers combined with plasticizer increased the air content in fresh concrete (6.4%) of concrete mixture CEM I - GR-PF (Table 2).

The amount of mixing water was almost identical in all concrete mixtures with the exception of CEM I - GR-PF. Firstly, dose of water of this concrete mixture had to be increased to achieve optimal consistency of fresh concrete; the reason was higher dose of polypropylene dispersed reinforcement, which worsens consistency of fresh concrete [16]. Secondary effect of the increased amount of water was to achieve lower strength properties of the mixture. As a consequence of increased water-cement ratio, the porosity of the cement stone was increased and thus high humidity of the specimens was reached. Thus, a mix of different characteristics used for comparison was obtained. The same plasticizer (superplasticizer Mapefluid N200) and its dosage were used for concrete mixtures CEM III/B - B, CEM III/B - B-PF, and CEM III/B - B-SF. The same plasticizer (plasticizer ChrysoFluid Optima 206) was used for concrete mixtures CEM I - GR-PF, CEM I - GR-SCC; however, the dosing was different. The dose of plasticizer was lower for a mixture CEM I - GR-PF due to an increased amount of water and thanks to this we wanted to achieve lower strength while maintaining an acceptable consistency. On the contrary, the amount of plasticizer for CEM I - GR-SCC was considerably increased so that rheological properties of fresh concrete could correspond to the properties of SCC. For the same reason the addition of fly ash was dosed for CEM I - GR-SCC; fly ash was produced in a power plant with classic high temperature combustion of anthracite.

Labeling, characteristics, and results for selection of individual mixtures are as follows.

*Mixture CEM I - GR-PF*:

- use of ordinary Portland cement,
- use of coarse aggregate, granodiorite,
- increased dose of dispersed polypropylene reinforcement,
- achieving of lower strengths of hardened concrete.

*Mixture CEM III/B - B*:

- use of blast-furnace cement,
- use of coarse aggregate, basalt,
- reference mixture for mixtures CEM III/B - B-PF, CEM III/B - B-SF.

*Mixture CEM III/B - B-PF, CEM III/B - B-SF*:

- use of blast-furnace cement,
- use of coarse aggregate, basalt,
- use of various dosages of various types of dispersed reinforcement.

*Mixture CEM I - GR-SCC*:

- use of ordinary Portland cement,
- use of coarse aggregate, granodiorite,
- designed as self-compacting concrete (increased proportion of fines, increased amount of plasticizer),
- specimens prepared without vibrating.

Properties of concrete in fresh state were determined in accordance with the following procedures:

 EN 12 350-2 testing fresh concrete, Part 2: slump-test [17],
 EN 12350-8 testing fresh concrete, Part 8: self-compacting concrete, slump-flow test [18],
 EN 12350-10 testing fresh concrete, Part 10: self-compacting concrete, L-box test [19],
 EN 12 350-6 testing fresh concrete, Part 6: density [20]
 EN 12 350-7 testing fresh concrete, Part 7: air content, Pressure methods [21].

### 2.2. Process of Experimental Work

Prior to thermal load, all sets of testing specimens were stored in humid area with constant moisture ±80% and constant temperature ±20°C. Thermal load with direct flame was carried out with gas burner Rothenberg, which can develop a flame with temperature up to 1300°C. The aim of thermal exposition was achieving high thermal gradient at the beginning of the exposition until the moment of achieving the temperature of 1200°C. To achieve this extreme thermal load, the development of temperature of flame was regulated so that the thermal curve was as close as possible to the hydrocarbon fire curve [22], however, only for the time of 60 minutes. Time of thermal load was reduced because of smaller size of test specimens.

**Figure 1:**
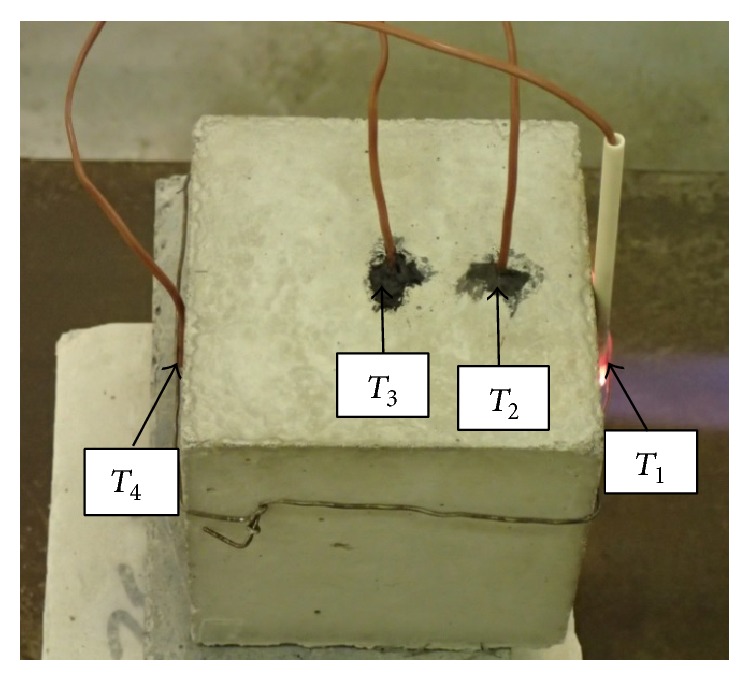
Location of individual thermocouples.

Temperature of thermal load and development of temperature in the specimens were detected by four thermocouples *T*_1_,  *T*_2_, *T*_3_, and *T*_4_ labeled TT-K-24-SLE (K-type, material Cr-Al, temperature range 0°C–1250°C, accuracy ±1.1°C) and recorded in the apparatus Kimo HD 200 (four channel modules with thermocouple sensors). Thermocouples were located on the exposed surface, in various depths from the heated surface and on the surface opposite to the exposed one (Figure 1):

(*T*_1_) thermocouple in the middle of the specimen, exposed surface (wiring of the thermocouple insulated in heat-resistant ceramic tube),
(*T*_2_) thermocouple in the middle of the specimen, at the distance of 25 mm from the exposed surface,
(*T*_3_) thermocouple in the middle of the specimen, at the distance of 50 mm from the exposed surface,
(*T*_4_) thermocouple in the middle of the specimen, opposite surface to the exposed one.

## 3. Results of Individual Tests and Discussion

### 3.1. Physical-Mechanical Properties before Heating

All test specimens were analyzed before exposition to thermal load; physicomechanical properties were determined (density, compressive strength, and flexural strength). When the specimens were thermally loaded, other test specimens from the same concrete mixtures were placed into drying kiln to determine current level of moisture of tested specimens. Properties of individual sets of test specimens before exposition to direct flame are given in Table 3.

Properties of concrete in hardened state were determined in accordance with following procedures:

 EN 12 390-3 testing hardened concrete, Part 3: compressive strength of test specimens [23],
 EN 12 390-5 testing hardened concrete, Part 5: flexural strength of test specimens [24],
 EN 12 390-7 testing hardened concrete, Part 7: density of hardened concrete [25]
 ČSN 73 1316 determination of moisture content, absorptivity, and capillarity of concrete [26].

### 3.2. Observations during Heating

Test specimens were visually observed during heating and changes were recorded; records are stated in Table 4 and details are in Figure 10. Exposed surface was photographically documented before thermal load and at 10-minute intervals. Development of temperature of the specimens was documented with thermocamera (see Figure 9).

After heating with direct flame, test specimens were always placed in common laboratory environment until they cooled down. Then, analysis of individual test specimens was carried out:

- visual observation of the specimen (description given in Table 5);
- observation of the network of cracks (cracks highlighted with a marker) and photographed (see Figures 7 and 8);
- measuring maximal depth of concrete spalled off by means of calibrated calipers if explosive spalling occurred (stated in Table 5).

**Figure 2:**
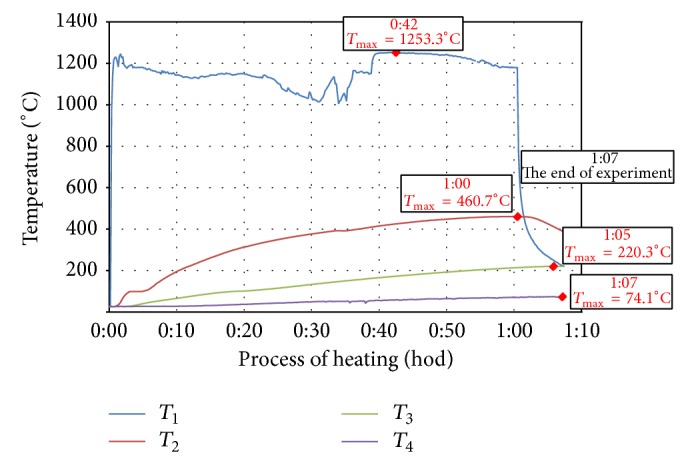
Record of thermal development of the test specimen CEM I - GR-PF.

**Figure 3 fig3:**
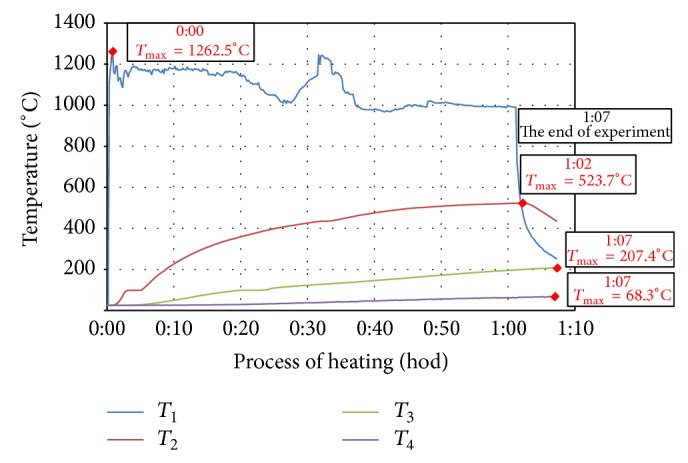
Record of thermal development of the test specimen CEM III/B - B-PF.

**Figure 4 fig4:**
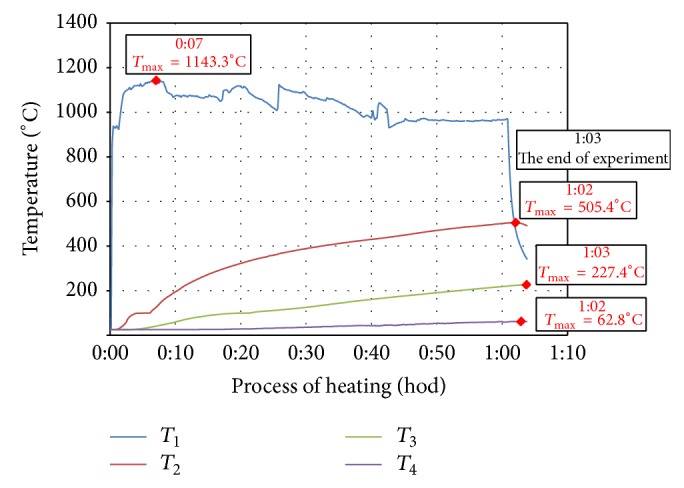
Record of thermal development of the test specimen CEM III/B - B-SF.

**Figure 5 fig5:**
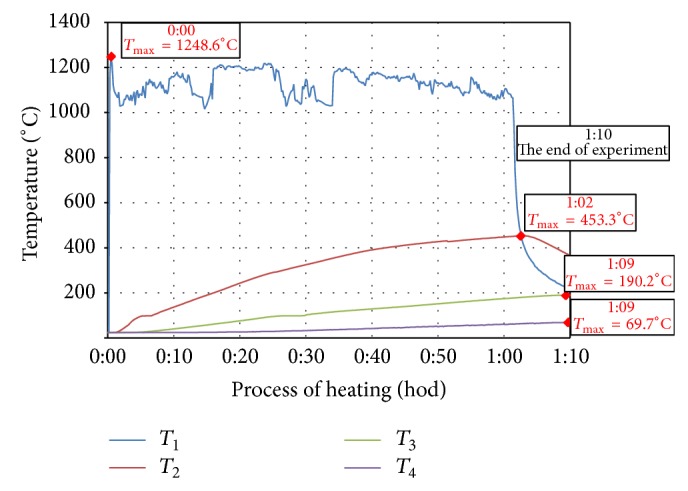
Record of thermal development of the test specimen CEM III/B - B.

**Figure 6:**
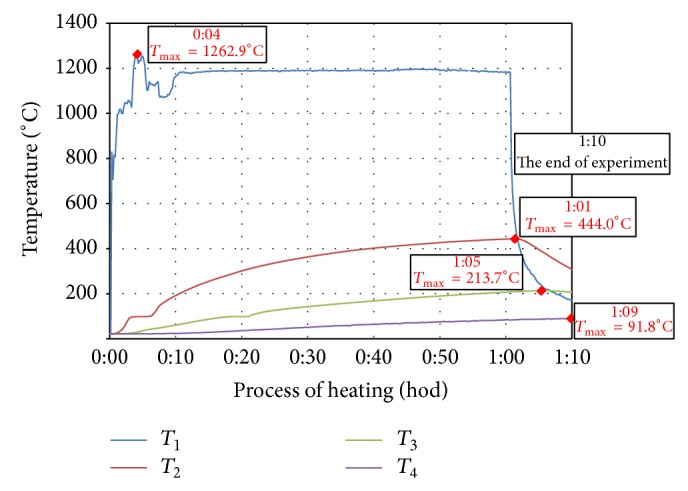
Record of thermal development of the test specimen CEM I - GR-SCC.

**Figure 7:**
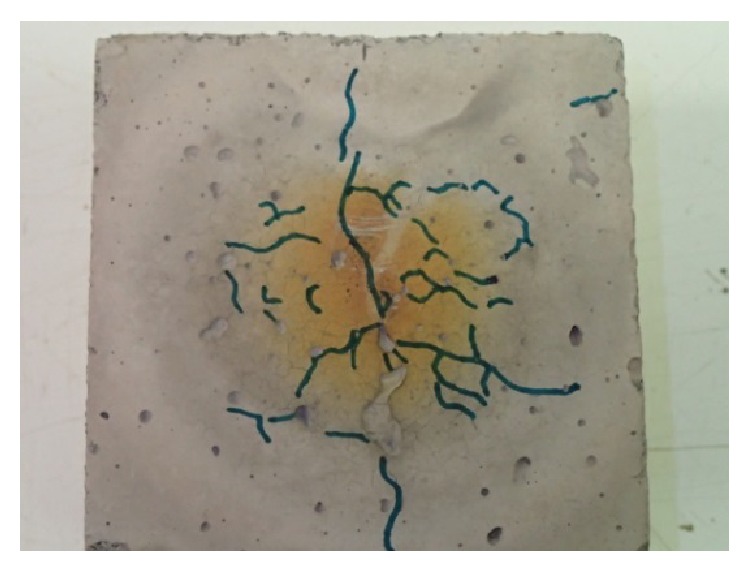
Detail of surface after heating with marked cracks, specimen CEM III/B - B-PF.

**Figure 8:**
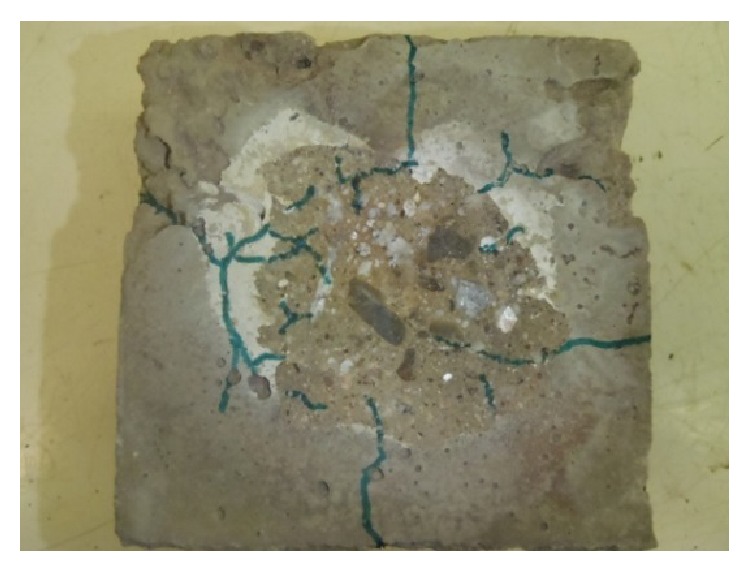
Detail of surface after heating with marked cracks, specimen CEM I - GR-SCC.

**Figure 9:**
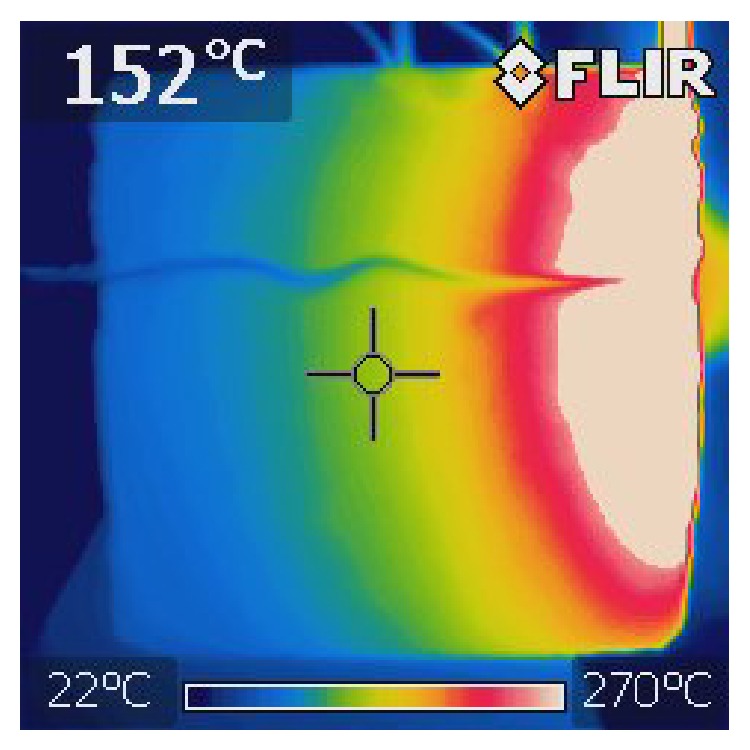
Thermophotograph immediately after the test, specimen CEM I - GR-SCC.

**Figure 10:**
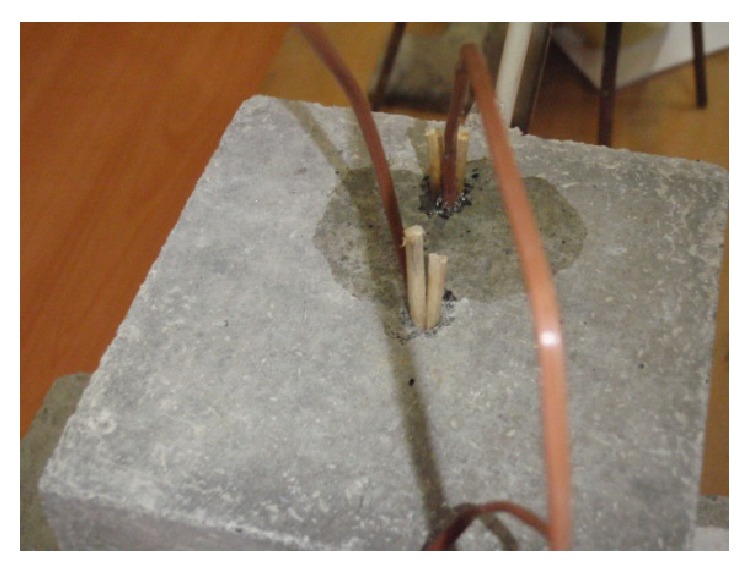
Detail of escaping moisture at the *T*_2_ location, specimen CEM I - GR-SCC.

Measured values of temperature development of individual test specimens during heating, which were detected with thermocouples and recorded in the recording module, are demonstrated in Figures 2 [Fig fig3] [Fig fig4] [Fig fig5]–6. In each curve of temperature measured with thermocouples (*T*_1_, *T*_2_, *T*_3_, and *T*_4_) there is a red point denoting maximal temperature *T*_max⁡_ (°C), which is stated in the frame above the red point as well as the time, when the maximal temperature was achieved (hours:minutes) from the beginning of thermal exposition.

Curves of thermal load of tested specimens, measured on the surface of the specimen (*T*_1_), showed differences up to 200°C because of manual regulation of the gas burner. Maximal temperatures measured by *T*_1_ (surface of specimen) were 1140°C–1260°C, *T*_2_ (25 mm below exposed surface) 444°C–524°C, *T*_3_ (50 mm below exposed surface) 190°C–227°C, and *T*_4_ (100 mm far from exposed surface) 63°C–92°C.

No major failures of concrete were observed during heating, with the exception of the mixture CEM I - GR-SCC. Specimens made from mixtures CEM I - GR-PF, CEM III/B - B, CEM III/B - B-PF, and CEM III/B - B-SF showed escape of moisture from side walls, most significant on the specimen CEM I - GR-PF (highest moisture content 4.4%). Specimen from mixture CEM I - GR-SCC showed explosive spalling of a layer of concrete from exposed surface after approximately 60 seconds from beginning of the experiment (current temperature ±1000°C). After another 60 seconds, a second wave of spalling occurred. After that, only escape of moisture from side walls (water) and extensive escape of moisture (water + water vapor) from the top wall of the specimen, where *T*_2_ thermocouple was fixed, were observed.

Specimen from mixture CEM III/B - B-PF (moisture content 4.1%) showed the least extent of failures; network of cracks on the exposed surface was apparent. The highest extent of failures was observed at the specimen from mixture CEM I - GR-SCC (moisture content 4.2%), where explosive spalling occurred on the exposed surface and maximal measured depth was 5.1 mm. These observations confirm positive effect of polypropylene fibers combined with thermally stabile basalt aggregate, specimen from mixture CEM III/B - B-PF. Spalling of specimen from mixture CEM I - GR-SCC occurred because of high density of structure (high dose of cement + addition), which prevented escape of heated water vapor. As a consequence, a layer of concrete was blasted off at the depth, where overheated water vapor gathered with no escape way. Specimens made from other mixtures (CEM I - GR-PF, CEM III/B - B, and CEM III/B - B-SF) showed failures in the form of network of cracks on the exposed surface and cracks in side walls.

## 4. Conclusion

The aim of experimental work was verification of behavior of concrete test specimens exposed to direct flame, which can closely simulate behavior of concrete structure at the conditions of real fire. Development of acting temperatures of the gas burner was regulated so that the curve of temperatures actin on the surface of the test specimen (where the *T*_1_ thermocouple was located) was close to hydrocarbon fire curve [22]. Temperature of this curve develops so that the temperature is around 1000°C after 10 minutes and after 20 minutes the temperature is around 1100°C. Deviations of from hydrocarbon thermal curve in our real experiment were caused by manual regulation of gas burner. Development of temperature was faster in the experiment; temperature on the surface of the test specimen (location of *T*_1_ thermocouple) was in most cases around 1200°C after 1 minute.

During exposure of specimens CEM I - GR-PF, CEM III/B - B, CEM III/B - B-PF, and CEM III/B - B-SF the escape of moisture from the side walls was observed, mostly in specimen CEM I - GR-PF (because of the high moisture content 4.4%).

On the specimen CEM I - GR-SCC an explosive spalling of the concrete layer from the exposed surface occurred already after about 60 seconds of the experiment (actual temperature ±1000°C). After another 60 seconds, the second wave of spalling occurred. Then, only the escape of moisture from the side walls (water) and a large leakage of moisture (water + steam) from the top wall of the specimen at the point thermocouple *T*_2_ were observed. The greatest amount of failures occurred in specimen CEM I - GR-SCC, where there was spalling from the exposed surface layer to a maximum depth of 5.1 mm observed. The smallest amount of failures occurred on the specimen CEM III/B - B-PF (moisture 4.1%); there was visible network of cracks only in the exposed surface. On other specimens (CEM I - GR-PF, CEM III/B - B, and CEM III/B - B-SF), failures in the form of networks of cracks in the exposed surface and cracks in the side walls were observed after the experiments.

The experimental work brought many observations and new information and data about behavior of concrete specimens of various compositions and strengths thermally loaded by direct flame; the most important ones are below.

- It was confirmed that use of fine polypropylene dispersed reinforcement prevents explosive spalling of concrete thanks to “opening” the structure of cement matrix by melting off (mixtures CEM I - GR-PF, CEM III/B - B-PF). As for the effect of addition of polypropylene fibres in mixture CEM I - GR-PF, it must be mentioned, that the higher content of mixing water caused the higher porosity (air content in fresh concrete CEM I - GR-PF was 6,4%). The better spalling behaviour of CEM I - GR-PF mixture was not only due to addition of PP fibre. The positive effect was reached by combination of the increased porosity and addition of polypropylene fibres.
- Specimen with fine steel fiber dispersed reinforcement (CEM III/B - B-SF) also showed no explosive spalling on exposed surface. Positive effect of these fibers lies in transferring tension caused by gathered overheated water vapor in concrete structure, which prevents explosive spalling.
- Fundamental failure of specimen CEM I - GR-SCC (explosive spalling) occurred in the first minutes of the experiment (within 3 minutes from beginning of the experiment).
- Test specimens CEM III/B - B and CEM I - GR-SCC had no dispersed reinforcement and comparable moisture content (±4%); explosive spalling occurred only on the test specimen with higher density of structure (CEM I - GR-SCC) in consequence of higher proportion of fines.

The nondestructive monitoring of the effect of high temperature on concrete specimens of different mixtures was performed. As an important fact to evaluate the resistance of concrete to high temperatures occurs a continuous evaluation of concrete behavior over time when exposed to high temperatures. The photographing of the surface of the specimens at intervals of 10 minutes was performed. Recording of significant behavioral changes of concrete was conducted throughout the experiment. In particular, it is important to record the time interval when explosive spalling occurs. This must be done in order to ensure protection of steel reinforcement in concrete and the possible prediction of structural failure in case of detection of reinforcement due explosive spalling and for the time required for evacuation. Using other methods of nondestructive testing makes it possible to spread information about the processes inside the thermally loaded specimen.

The following nondestructive methods for determination of changes and defects inside the specimens after thermal exposition can be used: determination of velocity of propagation of ultrasonic waver, X-ray tomography. As a next step, these tests can be complemented with destructive testing: determination of physicomechanical properties, X-ray, DTA, and porosimetry.

Presented results contribute to knowledge about behavior of real concrete structure thermally loaded by direct flame, a fire, which is currently very topical. The paper is valuable for setting the method of testing concrete specimen exposed to direct flame, which proved to be successful. This way of testing concrete specimens of smaller size is flexible, with low requirements for time and resources and it is a suitable early stage for more costly thermal loading of large scale concrete specimens with direct flame.

## Figures and Tables

**Table 1:** Properties of dispersed reinforcement used [10, 11].

Type of fibers	Fibrin 615	Fibrin 3/15	CAR25CDM

Material of fiber	Polypropylene	Polypropylene	Steel
Density	910 kg/m^3^	910 kg/m^3^	7 860 kg/m^3^
Length of fiber	6 mm	12 mm	25 mm
Diameter of fiber	5 *μ*m	16 *μ*m	500 *μ*m
Melting point	150–160°C	160°C	1 515°C

**Table 2:** Concrete mixtures of tested specimens and their properties in fresh state.

Component [kg/m^3^]/mixture	CEM I - GR-PF	CEM III/B - B	CEM III/B - B-PF	CEM III/B - B-SF	CEM I - GR-SCC
CEM I 42.5 R	350	—	—	—	360
CEM III/B 32.5 N	—	350	350	350	—
Mined aggregate 0/4 mm (spar gravelous sand)	894	—	—	—	930
Mined aggregate 4/8 mm (spar gravelous sand)	—	—	—	—	200
Coarse aggregate 8/16 mm (granodiorite)	928	—	—	—	510
Coarse aggregate 0/4 mm (basalt)	—	1 070	1 070	1 070	—
Coarse aggregate 4/8 mm (basalt)	—	1 070	1 070	1 070	—
Water	194	175	175	175	174
Plasticizer ChrysoFluid Optima 206	2.3	—	—	—	5.5
Superplasticizer Mapefluid N200	—	3.5	3.5	3.5	—
Fly ash	—	—	—	—	90
Polypropylene fibers Fibrin 615	2.0	—	—	—	—
Polypropylene fibers Fibrin 3/15	—	—	1	—	—
Steel fibers CAR25CDM	—	—	—	50	—
Consistency (slump test) [mm]	150	130	30	80	—
Consistency (slump-flow test) [mm]	—	—	—	—	670
Time *T*_500_ (slump-flow test) [s]	—	—	—	—	5.0
Time *T*_400_ (L-box test) [s]	—	—	—	—	6.5
*H*_2_/*H*_1_ (L-box test) [—]	—	—	—	—	0.97
Air content in fresh concrete [%]	6.4	2.2	3.4	2.5	1.8
Density of hardened concrete [kg/m^3^]	2 180	2 560	2 550	2 610	2 360

**Table 3:** Physical-mechanical properties of samples before heating.

Mixture	Density [kg/m^3^]	Compressive strength [N·mm^−2^]	Flexural strength [N·mm^−2^]	Moisture [%]
CEM I - GR-PF	2 010	21.5	4.2	4.4
CEM III/B - B-PF	2 450	37.7	6.2	4.1
CEM III/B - B-SF	2 500	27.7	5.6	2.9
CEM III/B - B	2 620	33.9	6.4	3.7
CEM I - GR-SCC	2 280	56.6	8.1	4.2

**Table 4:** Description of individual specimens during heating.

Mixture	Exposure time	Description

CEM I - GR-PF	7 min	First crack formed on the side with thermocouples, where moisture escapes

CEM III/B - B-PF	—	With no outstanding changes during heating

CEM III/B - B-SF	—	With no outstanding changes during heating

CEM III/B - B	—	With no outstanding changes during heating

CEM I - GR-SCC	1 min	Explosive spalling (depth approx. 5 mm)
	2 min	Another explosive spalling
	4 min	Escape of moisture from side walls of the specimen, extensive escape of moisture and water vapor where the second thermocouple is fixed

**Table 5:** Description of test specimens after thermal load.

Mixture	Spalling [mm]	Description
CEM I - GR-PF	Nothing	(i) Network of cracks on the exposed surface, local cracks in side walls
CEM III/B - B-PF	Nothing	(i) Network of cracks on exposed surface
CEM III/B - B-SF	Nothing	(i) Network of cracks on exposed surface, perpendicular crack on the surface with thermocouples
CEM III/B - B	Nothing	(i) Network of cracks on exposed surface, perpendicular crack on the surface with thermocouples
CEM I - GR-SCC	5.1	(i) Spalling of a layer of concrete from the exposed side (ii) Local cracks in side walls (iii) Cracks in mastic cement and aggregate of spalled surface
